# Seasonal vaccines – Critical path to pandemic influenza response

**DOI:** 10.1016/j.vaccine.2016.12.056

**Published:** 2017-02-07

**Authors:** Wenqing Zhang, Siddhivinayak Hirve, Marie-Paule Kieny

**Affiliations:** a Global Influenza Program, World Health Organization, Geneva, Switzerland; b Health Systems and Innovation, World Health Organization, Geneva, Switzerland

Influenza vaccine is the primary intervention to reduce mortality and morbidity in an influenza pandemic, in particular a severe pandemic. However, the current state of vaccine technology does not allow a rapid surge in pandemic vaccine production in the advent of a pandemic, to which the response will rely on the same production platform as seasonal vaccines. Thus how efficient a pandemic vaccine response would be depends largely on the extent of how well seasonal vaccine response to annual epidemics is rightly seized. Recognizing the shared pathways of vaccine response to both seasonal epidemics and pandemics, in 2002 WHO developed a “Global Agenda on Influenza Surveillance and Control” with an objective to increase influenza vaccine usage during inter-pandemic periods towards strengthen national and international pandemic preparedness. In 2003, the World Health Assembly (WHA 56.19) resolved to urge Member States to increase vaccination coverage of risk population [1]. In 2006, the Global Action Plan (GAP) on Influenza Vaccines was launched to promote evidence-based seasonal vaccine use, increase vaccine production capacity and stimulate research and development [2].

With the past decade’s efforts of WHO and its partners, the accumulated knowledge, understanding, experience and practice of influenza vaccines results in a renewed landscape of progress and challenges. Among others, vaccine research and development led by US Biomedical Advanced Research and Development Authority (BARDA) and increased potential vaccine production capacity pioneered by the WHO GAP have resulted in a far better pandemic preparedness [3], [4]. On the other hand, the need for thorough preparedness as learnt from the recent Ebola outbreak response [5] mirrors that for an influenza pandemic, including pandemic vaccine response. The most opportunistic estimate of current monovalent pandemic vaccine production capacity of 415 million doses per year in GAP-supported Low- and Lower-middle-income countries (GAP survey 2015 – unpublished) is far from the need to immunize the world’s population of more than 7 billion people, let alone that pandemic influenza waves may have swept over globally within a year. Aside from the supply, the current 50–60% effectiveness of seasonal vaccines, even with perfect match of vaccine viruses with circulating viruses [6], is rather disappointing and calls for better protective vaccines. The perception on influenza vaccines is further affected when there is a mismatch between vaccine composition and circulating viruses, which can result in effectiveness much lower than 50%. The scientific complexities e.g. issues associated with egg-propagated and cell-propagated virus isolates, and newly emerged viral features of seasonal H3N2 viruses [7], [8] which is not fully comprehended by growing interest of the population, press the challenge further.

The relatively mild severity of the 2009 H1N1 pandemic [9] does not in any way exempt the world from a catastrophic hit at any time by the next pandemic strain of an hitherto unknown virus subtype. The threat of the next influenza pandemic is getting more vivid than ever with the spreading of avian influenza viruses [10], and increasing virus activity across the animal human ecosystem interface [11], [12], of which, tip of the iceberg can be seen from the frequent as yet sporadic and extremely alarming human infections with H5, H7N9 and H9N2 avian viruses. WHO revised its pandemic preparedness plan and published an interim guidance document of Pandemic Influenza Risk Management Framework in 2013 [13], and is currently working with partners towards finalizing the pending chapter on pandemic vaccines.

The complexities of vaccine response at the beginning of a pandemic were examined through an informal WHO consultation in June 2015 through scenarios, when seasonal influenza viruses may still be causing severe epidemics in parts of the world. Subsequently countries may have different priority needs for seasonal and pandemic vaccines, while a vaccine manufacturer can produce at one time either seasonal or pandemic vaccines but not both. The WHO process continued in July 2016 (unpublished) to develop response with critical elements including principles of consideration and a transparent process for decision to commence the pandemic vaccine production which might require “switch” from seasonal vaccine production. Such decision making process will not be fully operational until critical details e.g. data needs as evidence base are identified and vaccine production bottleneck solutions are in place. Except for some conditional regulatory processes [14], [15], [16] specific to pandemic vaccines, all steps of pandemic vaccine production are aligned with those of seasonal influenza vaccines. This forms the base of the pandemic vaccine preparedness strategy.

Early detection of a pandemic virus and development of a high-growth reassortant that can be used for large scale production are critical steps of pandemic vaccine response. The constraints of these early critical steps are reflected in seasonal vaccine response – which became very evident when an antigenic variant emerged late in spring 2014 [17] leading to a mismatch of H3N2 component of the vaccines for use in the northern hemisphere 2014–15 season. Such events also call for a critical review of the resilience of current system from virus detection to vaccine production and distribution to deal with late emerging seasonal variants – the same mechanism for the pandemic vaccine response.

Since 1952, WHO through its Global Influenza Surveillance and Response System (GISRS) is working closely with its partners to further strengthen global surveillance, including the detection of novel antigenic variants and monitoring their spread – a decisive signal of a pandemic, as part of the seasonal vaccine response. The adoption and implementation of the Pandemic Influenza Preparedness (PIP) Framework [18] enables a systematic approach to understand burden of disease associated with influenza, which is essential for countries in particular in Low- and Lower-middle-income countries to shape their seasonal vaccination policies. Such vaccination policies will foster the development of national surveillance, regulatory and deployment capacity during the inter-pandemic periods to serve pandemic response preparedly.

Influenza vaccine production technology has remained largely the same since several decades ago, though new technologies such as synthetic technology have been used in emergency situations to develop candidate vaccine viruses as during the rapid vaccine response to the H7N9 outbreak [19]. For a world population of more than 7 billion currently with about 79% living in low and middle income countries, in the event of a severe pandemic, the only way to avoid a major global disaster is, from this moment on, to expedite research and development of new vaccines and vaccine production technologies in parallel with strengthening national and global surveillance, regulatory, logistics and communication capacities. This requires a multi-disciplinary effort from funders, public sector agencies, industry, national governments and academia. In this context, the WHO Global Influenza Program in Geneva has currently undertaken to update its research agenda first developed in 2009 and reviewed in 2011 [20], [21], through wide consultations with key stakeholders to channel global research efforts to areas most needed to shape risk mitigation and response optimization.

Ten years of experience will have been gained when the WHO Global Action Plan for Influenza Vaccines concludes in November 2016. Five years of the implementation of the PIP Framework has been reviewed [22] and will be discussed by the WHO Member States in January 2017. These provide an opportunity, with engagement of all key players on influenza vaccines, to closely evaluate the achievements and deliberate on critical gaps of pandemic response including the post-GAP deficiencies that should be addressed especially in the context of influenza vaccine production, access and use in the developing world.

The actual pandemic influenza vaccine response pathway, sharing many aspects with that of the seasonal vaccines, is subject to massive stress, extremely complex and dependent on many critical and concrete steps from detection of the pandemic virus, to vaccine production and roll-out. Aside from some response elements e.g. actual vaccine virus that would be available only when a pandemic hits, seasonal influenza – a robust vaccine process and better vaccines, provides a critical opportunity to maximize the efficiency and effectiveness of the pandemic vaccine response. Luck, as was the 2009 pandemic, is not a strategy. The inter-pandemic period now presents a precious window, but the window may close at any moment.
